# The Vestibular-Auditory Interaction for Auditory Brainstem Response to Low Frequencies

**DOI:** 10.1155/2014/103598

**Published:** 2014-03-31

**Authors:** Seyede Faranak Emami, Nasrin Gohari

**Affiliations:** Department of Audiology, Faculty of Rehabilitation, Hamadan University of Medical Sciences and Health Services, Hamadan 16657-696, Iran

## Abstract

Since saccular projection is sound sensitive, the objective is to investigate the possibility that the saccular projections may contribute to auditory brainstem response to 500 HZ tone burst (ABR_500 HZ_). During the case-control research, twenty healthy controls compared to forty selected case groups as having chronic and resistant BPPV were evaluated in the audiology department of Hamadan University of Medical Sciences (Hamadan, Iran). Assessment is comprised of audiologic examinations, cervical vestibular evoked myogenic potentials (cVEMPs), and ABR_500 HZ_. We found that forty affected ears of BPPV patients with decreased vestibular excitability as detected by abnormal cVEMPs had abnormal results in ABR_500 HZ_, whereas unaffected ears presented normal findings. Multiple comparisons of mean p13, n23 latencies, and peak-to-peak amplitudes between three groups (affected, unaffected, and healthy ears) were significant. In conclusion, the saccular nerves can be projective to auditory bundles and interact with auditory brainstem response to low frequencies. Combine the cVEMPs and ABR_500 HZ_ in battery approach tests of vestibular assessment and produce valuable data for judgment on the site of lesion. Regarding vestibular cooperation for making of wave V, it is reasonable that the term of ABR_500 HZ_ is not adequate and the new term or * vestibular-auditory brainstem response to 500 HZ tone burst *is more suitable.

## 1. Introduction

The evolutionary adaptations in the mammalian and the human inner ear allow selective activation of auditory or vestibular hair cells. The pars superior of the labyrinth (utricle and semicircular canals) has remained fairly constant throughout evolution, whereas the pars inferior (saccule and other otolith, macular, and auditory end organs) has seen considerable change as many adaptations were made for the development of auditory function [1]. The saccular projections are involved with the transduction of acoustic stimuli [2]. These fibres travel caudally through the descending vestibular nucleus, next are entered into the cochlear nucleus, and terminate at cells situated between the dorsal and posteroventral cochlear nucleus [3]. Some of them send projections from cochlear nucleus to various auditory fields [4]. The main portion of auditory brain (temporal gyrus) can activate from the vestibular sensitivity to sound [5], which is activated in response to stimuli that may be used clinically to evoke cVEMPs [5–7]. It provides a means of assessing saccular function and is elicited by the sound of low frequency and loud intensity within the range (100–1000 HZ) of human hearing [8].

**Figure 1:**
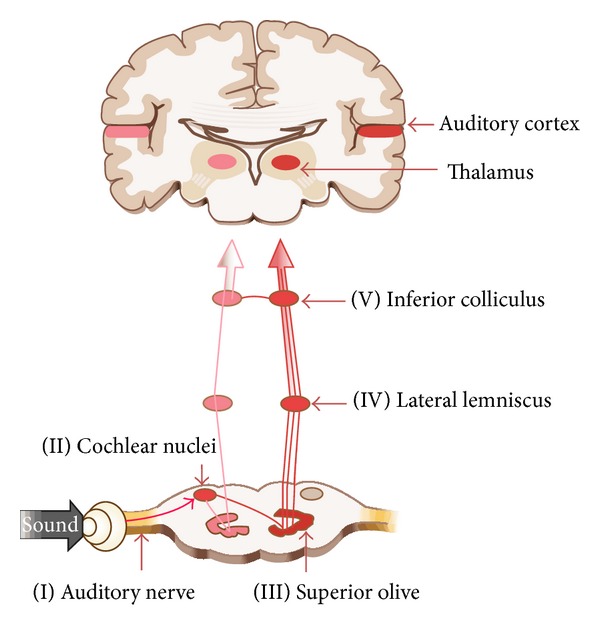
The neural generators of auditory brainstem response in afferent auditory pathway.

On the other hand, ABR_500 HZ_ provides an estimate of low frequency sensitivity, which is called slow-wave negative (SN10) response [9, 10]. The sharp peak (wave V) of ABR_500 HZ_ is generated by the lateral lemniscus as it terminates into the inferior colliculus and the activity of the inferior colliculus is responsible for the generation of the relatively slow and large negativity following the peak of the wave V (Figure 1) [11, 12]. For some individuals, the amplitude of the ABR_500 HZ_ can achieve several times of the faster (click) ABR component. Several recent studies have demonstrated strong relationships between ABR_500 HZ_ thresholds and pure-tone_500 HZ_ behavioral thresholds, with correlations of 0.9 and higher in subjects with normal and impaired hearing [13]. However, neurons at the brainstem and primary auditory cortex are responsive to the low frequency [11]. It is known that low frequency components are important contributors in the neural phenomena and may serve as the basis for hierarchical synchronization function through which the central nervous system processes and integrates sensory information [14]. Thus, the objective is to investigate the possibility that the saccular projections may contribute to ABR_500 HZ_.

## 2. Materials and Methods

The type of study is case control, which involved twenty volunteer healthy persons (11 females and 9 males; mean age: 31 years and range: 18–43 years = 40 ears) and forty selected case groups (24 females and 16 males; mean age: 30 years and range: 26–35 years = 80 ears) as having chronic and resistant benign paroxysmal positional vertigo (BPPV). The diagnosis of patients with BPPV found results of typical nystagmus (torsional upbeating nystagmus with latency and fatigue lasting less than 1 min and subjective vertigo in the Dix-Hallpike). They were not treated with canalith repositioning maneuver (CRM) on the side determined by Dix-Hallpike test (the repetition of CRM was five with intervals of 5 days) [15] and presented to Audiology Department of Hamadan University of Medical Sciences from April to June 2013.

The inclusion criteria involved BPPV disorder with normal function ofhearing, middle ear pressure, ipsilateral and contralateral acoustic reflexes, auditory brainstem responses, and abnormal cVEMPs.

*Assessment*. The calibration of our instruments (full set of LABAT evoked potential recorder, MADSEN diagnostic audiometer, HOMOTH impedancemetre) had been kept under control. Testing was performed bilaterally and consisted of pure tone audiometry, immittance measures, videonystagmography (VNG), click-evoked ABR, ABR_500 HZ_, and cVEMPs. All of the tests were performed on the same day.

Hearing thresholds in the normal range (−10 to 15 dB_HL_) were obtained from each person over the frequency range of 250–8000 Hz [16]. Impedance measures consisted of normal tympanometry (between ±50 dapa) [17] and acoustic values (85 to 100 dB_Spl_) [18]. VNG was used to eliminate the possibility of any additional vestibular pathology (saccade, tracking, optokinetic, Dix-Hallpike, and caloric tests) [15]. Click-evoked auditory brainstem response provides an estimation of high frequency (2000–4000 HZ) sensitivity [19]. The response was abnormal, when peaks III and/or V were absent or when the peak-to-peak I-V exceeded the normal limits of our laboratory (4.40 ms for females, 4.58 ms for males).

Auditory brainstem response to 500 HZ tone burst (ABR_500 HZ_): the patients were placed in the supine position on a gurney within a sound-treated room. Noninverting electrode was placed at the high forehead and inverting electrode on ipsilateral mastoid and ground electrode on contra lateral. Electrode impedances were roughly equivalent and were < 5 kilohms at the start of the test. Responses to 2000 stimuli were averaged (rate of 37/s, filtered from 30 to 3000 HZ, 2-0-2, 500 HZ tone burst, 120 dB_SPL_; contralateral noise = 90 dB_SPL_, window of 25 ms) [11, 12]. The ABR_500 HZ_ concluded to be abnormal, when wave V was absent or when it exceeded the normal limits of our laboratory.

Cervical vestibular evoked myogenic potentials (cVEMPs): during cVEMPs (air-conducted) recording patients were instructed to turn and hold their heads as far as possible toward the side contralateral to the stimulated ear. At that point, the overall electromyogenic activity of the sternocleidomastoid muscle (SCM) was set as the reference level of the tonic contraction. Patients were asked to maintain contraction at this level throughout the test session (approximately 50 s) [4]. The active electrode was placed over the middle portion of the ipsilateral SCM body. The reference and the ground electrodes were placed over the upper sternum and on the midline forehead, respectively. Auditory stimuli consisted of tone burst (500 HZ, 120 dB_SPL_, rise/fall time = 1 ms, plateau = 2 ms, grand-average = 200, and filtered = 20–20000 HZ) which presented to the ear ipsilateral to the contracted SCM [7]. The cVEMPs results for the healthy ears are used as normative data, and latencies and any cVEMPs asymmetry ratio (100 [(An − Ad)/(An + Ad)], An = p13 − n23 (the peak-to-peak amplitude in the normal ear), and Ad = p13 − n23 (the peak-to-peak amplitude in the affected ear) [4]) above the calculated upper limit are interpreted as abnormal.

*Data Analysis*. All analysis was done by means of the statistics software SPSS_17_. Data were expressed as mean ± standard deviation. Kolmogorov-Smirnov test was used for evaluation of normal test distribution. One-way ANOVA was used to compare findings among the three groups (case group = affected and unaffected ears, control group = healthy ears). Tukey's least significant difference (Tukey's HSD) test was chosen as the post hoc test. Also, *P* value of < 0.05 was considered to indicate statistical significance.

## 3. Results

In this study, we evaluated twenty healthy controls compared to forty selected case groups as having chronic and resistant BPPV. The latency and amplitude values of the cVEMPs were detectable in all healthy persons (Table 1). The case group had unilateral vestibulopathic ears (*n* = 40). Vestibulopathic ears had both decreased amplitudes and delayed latencies of the cVEMPs (affected) in thirty-five and absent responses in five. Unvestibulopathic ears (*n* = 40) presented normal cVEMPs findings (unaffected).

**Figure 2:**
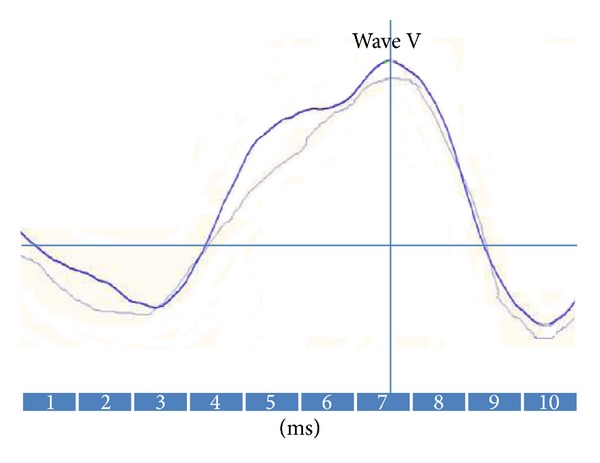
The auditory brainstem response to 500 HZ tone burst (ABR_500 HZ_) in a healthy ear.

ABR_500 HZ_ was recordable from all healthy persons and unaffected ears (Figure 2). But it only had lower amplitudes and longer latencies values in affected ears (Table 2). Multiple comparisons of mean p13, n23 latencies and peak-to-peak amplitudes between three groups (affected, unaffected, and healthy ears) were significant (*P* = 0.000 for all, one-way ANOVA test). Comparisons of mean p13, n23 latencies and peak-to-peak amplitudes in affected ears versus healthy group were significant (*P* = 0.000, Tukey's HSD). Since affected and unaffected ears belong to the same individuals (matched ears), so there would be no differences between these two regarding sex or age. Affected ears had both abnormal cVEMPs and ABR_500 HZ_ results, but unaffected ears presented normal findings.

## 4. Discussion

Forty affected ears of the case group with decreased vestibular excitability as detected by abnormal cVEMPs had distorted ABR_500 HZ_, whereas both unaffected and control ears presented normal results. Since the ABR_500 HZ_ and the cVEMPs are evoked by low frequency sound [4, 11], the saccular nerves may enter into afferent auditory pathway and the acoustic sensitivity of the saccule can improve the ABR_500 HZ_ response and may be effective in making the shorter latency, higher amplitude, and sharp shape of the waveform, while lower amplitude, rounded shape, and longer latency of auditory brainstem activity may occur in abnormal function of the saccule, which cannot transmit low frequency neural response from vestibular endings to cochlear nucleus. Then, the present study is a description of the vestibular-auditory interaction for auditory brainstem response.

Similarly, Burian and Gstoettner reported that the afferent saccular fibres (guinea pig) are involved with the transduction of acoustic stimuli. They found the superior branch of the vestibular nerve and the saccular projections run into the cochlear nucleus. These axons travel caudally through the descending vestibular nucleus, enter the cochlear nucleus at a level caudal to subgroup *y*, and terminate at cells situated between the dorsal and posteroventral cochlear nucleus [2].

Most of the saccular afferents however showed irregular spontaneous firing. The vestibular nucleus neurons in the lateral vestibular nucleus and in the rostral portion of the inferior vestibular nucleus are sound sensitive [4], and there is anatomical evidence of the projection from the vestibular nerves (mainly saccule) to the cochlear nucleus [7]. Some of the vestibular afferent nerves send projections to various auditory fields on the cortex, [5] and the areas of the humans auditory brain activate by vestibular sensitivity to sound [5, 6].

The people with normal saccular function have intact projections to cochlear nucleus, lateral lemniscus, and inferior colliculus [20]. These projections can increase the peaks of the ABR_500 HZ_ and increase neural response velocity. Consequently, lower amplitude, rounded shape, and longer latency of ABR_500 HZ_ are the signs of the absence of transmitted neural signals by saccular projections into auditory brainstem pathway.

On the other hand, the amplitude of an ABR component depends on the amount of neural conduction time, and the auditory fibers' responses have the best conduction time in the low frequencies [10]. The auditory fibers in the brainstem pathway are temporally precise, with better neural conduction time to F0 (fundamental frequency or lowest component of the sound). Indeed, the temporal pattern of fibers' responses in the auditory nerve and the cochlear nucleus to medial geniculate body are near-periodic, and the frequency of their repetition is synchronized with F0 [21]. The range of saccular sensitivity happens to coincide with the range of our voice pitch (F0 = *∼*100 up to 400 Hz) [8]. Pitch perception is crucial for music-speech perception and auditory object recognition in a complex acoustic environment, which conveys the phonetic information and prosodic cues such as intonation, stress (in European languages), and semantic information in tonal languages (Chinese, Vietnamese, and Thai) [20].

In addition, our recent study shows that percussive daf musical sound (daf is a Persian percussive instrument, which had important usage specially in celebrations) seems to be related to both saccular and cochlear dysfunction. The associated degeneration in the cochlear and saccular afferents is associated with the exposure to low frequency, high intensity percussive daf music. It may reflect their common sound sensitive function [22].

Recent studies show that the saccule is the site of phonetic self-regulating mechanism with positive feedback [6]. Low frequency cues of the sound spectrum have very important roles in auditory function, which can stimulate the saccular afferents [23]. Also, the sensation of the sound at low frequencies may be present in patients with total deafness and normal vestibular function (predominantly saccule). This improvement disappears when saccular function is lost [24]. In the case of healthy hearing human and in presence of severe competing noise, saccule has a facilitating role in cochlea and can improve detection of loud low frequencies [25]. Consequently, it may be valuable for speech processing and perception-production system [8].

However, the auditory brainstem is sensitive to the azimuth and elevation of sound source locations [14], and the brainstem's response to speech is the representation of the F0 [21]. Recent studies have shown in normal hearing that the saccular stimulation can activate cortical multisensory areas especially in the temporoinsular and temporoparietal cortex in both hemispheres [26].

After all, the acoustic sensitivity of the saccule to low frequency component is effective in neural activities; it can improve and contribute to auditory brainstem response. The ABR_500 HZ_ has a prediction role in saccular function and the observation of any abnormality in waveform pattern in presence of normal hearing can be a sign of damage to saccular hearing. So there is a vestibular-auditory interaction not only for hearing but also for auditory brainstem response to low frequencies. In battery approach test of vestibular assessment, the use of the cVEMPs and ABR_500 HZ_ can produce valuable data for judgment on the site of lesion. Regarding vestibular cooperation for making of wave V, it is reasonable that the term of ABR_500 HZ_ is not adequate and the new term or vestibular-auditory brainstem response to 500 HZ tone burst is more suitable.

## Figures and Tables

**Table 1:** The mean of the right and left latencies and amplitudes of cervical vestibular evoked myogenic potentials (cVEMPs) in the healthy ears and the affected ears of the dizzy patients.

Variable	Affected ears	Unaffected ears	Healthy ears
p13 (ms)	19.5 ± 1.4	12.85 ± 2.1	13.37 ± 1.9
n23 (ms)	26.45 ± 1.5	20.48 ± 1.8	19.56 ± 2.5
Peak-to-peak amplitude (µv)	37.08 ± 11.7	48.21 ± 32.3	45.57 ± 38.6

**Table 2:** The mean of the right and left latencies and amplitudes of auditory brainstem response to 500 HZ tone burst (ABR_500 HZ_) in the healthy ears and the affected ears of the dizzy patients.

Variable	Affected ears	Unaffected ears	Healthy ears
Peak-to-peak amplitude (µv)	0.6 ± 0.3	0.97 ± 0.48	1.09 ± 0.62
Latency (ms)	6.64 ± 0.67	5.84 ± 0.36	5.95 ± 0.57

## Abbreviations

(cVEMPs): Cervical vestibular evoked myogenic p
(ABR_500 HZ_): Auditory brainstem response to 500 HZ tone burst.
